# Investigation of the impact of total sleep deprivation at home on the number of intrusive memories to an analogue trauma

**DOI:** 10.1038/s41398-019-0403-z

**Published:** 2019-02-27

**Authors:** Kate Porcheret, Dalena van Heugten–van der Kloet, Guy M. Goodwin, Russell G. Foster, Katharina Wulff, Emily A. Holmes

**Affiliations:** 1 Sleep and Circadian Neuroscience Institute, Nuffield Department of Clinical Neurosciences, University of Oxford, Oxford, OX1 3RE UK; 2 Department of Clinical Psychological Science, Faculty of Psychology & Neuroscience, Maastricht University, Maastricht, 6200 MD NL Netherlands; 3 Department of Psychiatry, University of Oxford and Oxford Health NHS Foundation Trust, Oxford, OX3 7JX UK; 4 Departments of Radiation Sciences and Molecular Biology, Umeå universitet, Umeå, 901 87 SE Sweden; 5 Department of Psychology, Uppsala University, Uppsala, Sweden

## Abstract

Sleep enhances the consolidation of memory; however, this property of sleep may be detrimental in situations where memories of an event can lead to psychopathology, such as following a traumatic event. Intrusive memories of trauma are emotional memories that spring to mind involuntarily and are a core feature of post-traumatic stress disorder. Total sleep deprivation in a hospital setting on the first night after an analogue trauma (a trauma film) led to fewer intrusive memories compared to sleep as usual in one study. The current study aimed to test an extension of these findings: sleep deprivation under more naturalistic conditions—at home. Polysomnographic recordings show inconsistent sleep deprivation was achieved at home. Fewer intrusive memories were reported on day 1 after the trauma film in the sleep-deprived condition. On day 2 the opposite was found: more intrusive memories in the sleep-deprived condition. However, no significant differences were found with the removal of two participants with extreme values and no difference was found in the total number of intrusive memories reported in the week following the trauma film. Voluntary memory of the trauma film was found to be slightly impaired in the sleep deprivation condition. In conclusion, compared to our eariler findings using total sleep deprivation in a hospital setting, in the current study the use of inconsistent sleep deprivation at home does not replicate the pattern of results on reducing the number of intrusive memories. Considering the conditions under which sleep deprivation (naturalistic versus hospital) was achieved requires further examination.

## Introduction

It is commonly believed that a good night’s sleep is the best way to recover from a stressful day, and it is often assumed this will also be the case following highly distressing events such as psychological trauma. Common symptoms after a psychological trauma are intrusive memories of the traumatic event^1^. Intrusive memories are autobiographical^2^, emotionally tagged^3^ or mental imagery-based^4^ memories that reappear involuntarily in a person’s consciousness; i.e. they spring to mind spontaneously (compared to memories that are deliberately retrieved)^5^. Intrusive memories can be very distressing, distracting and debilitating, and form a core clinical feature of both acute stress disorder (ASD, when post-traumatic symptomatology occurs in the first month following a trauma^1^) and post-traumatic stress disorder (PTSD, when symptoms persist beyond the first month^1^). Early re-experiencing of a trauma in the first days after the event has been suggested to predict the longer-term development of ASD^6^ and PTSD^7,8^. A recent study of over 1000 patients following a traumatic injury concluded that ‘intrusive memories and reactivity are centrally associated with other symptoms in the acute phase [of PTSD symptoms], potentially pointing to the utility of addressing these symptoms in early intervention strategies’^9^. One strategy to target intrusive memories is to impair their initial consolidation. Early clinical studies using a cognitive task to compete with the memory consolidation of intrusive memory showed that compared to control this led to a reduction in the number of intrusive memories^10,11^, suggesting some support for early interventions for at least near-term effects. It is of course possible that early re-experiencing may predict pathology (identify a vulnerable individual) but not have any role in promoting pathology.

Sleep provides another domain through which to target intrusive memories. As sleep enhances the consolidation of emotional memory in most^12–16^, but not all studies^17^, it has been postulated that preventing sleep could interfere with the consolidation of intrusive memories^18^. Previously we have tested this hypothesis using the trauma film paradigm^19^, which involves participants actively viewing a series of distressing film clips (i.e. imagining themselves being at the scene, watching the events unfold), followed by 24 h of total sleep deprivation in a hospital setting^20^. We found that people who are fully sleep deprived on the first night immediately following the trauma film reported *fewer* intrusive memories in the first week (starting from the day after the trauma film), compared to a control ‘sleep as usual' group^20^. This lower number of intrusive memories was found for the first two days, along with lower psychological distress at the end of the first week, as measured by the Impact of Event Scale-Revised (IES-R)^21^. While this result is consistent with some neuroscience-guided hypotheses relating to memory consolidation^20^, these findings at first glance appear to contradict what is widely believed clinically regarding the general benefits of sleep after trauma (i.e. that poor sleep is assocaited with psychopathology).

Clinically, sleep disturbances have been reported to be *predictive* for the development of PTSD, as well as other psychiatric disorders, when self-reported both before^22^ and after^23^ a traumatic event, and when objectively assessed 1 month following the trauma^24,25^. Therefore the discrepancy with our experimental findings^20^, is a false polemic as clinical studies have focused on the weeks and months post truama, whereas we are focused on the first night of sleep only (i.e. the sleep episode where memories of the event undergo consolidation). In our previous study^20^, the sleep-deprived participants remained in hospital following the trauma, while the sleep group returned home to sleep. To assess both conditions in the same environment and to create a more naturalistic study, the aim of this study was to have both groups return home after the trauma film.

In Porcheret et al.^20^, the number of intrusive memories was assessed from the morning after the trauma film so that participants had the same time window to report intrusive memories in both conditions. Recently, James et al.^26^ reported the daily number of intrusive memories over 1 week immediately following a similar trauma film, in participants without sleep deprivation. The largest change in the number of intrusive memories was reported from day 0 (the day of the trauma film) to the next day^26^. To determine if the beneficial effect of extended wakefulness is driven by the lack of sleep (rather than an altered time course), intrusive memories need to be assessed from the time of the trauma film and throughout the sleep deprivation period.

Voluntary memory of a trauma film (assessed using verbal cued recall and recognition tasks) has not been found to affect the number of intrusive memories reported^27,28^. However, this is yet to be tested after sleep deprivation. Sleep is postulated to act differentially on the emotional component (operationalised as the emotional response to a reminder of the emotional event or stimulus) and the explicit core (i.e. the information about the event)^29^. Using a task based on the one used by Kuriyama et al.^30^, the present study aimed to investigate the impact of sleep deprivation on the emotional component of memory of the trauma film, with fear ratings for neutral images taken from the trauma film, compared to images of similar content but not from the trauma film. Responses were assessed before the trauma film, after the film and after one night of recovery sleep. Recognition and cued recall tasks were used to assess the explicit core.

In summary, the current study addressed the following hypotheses: (i) That, compared to sleep as usual at home, total sleep deprivation on the first night following analogue trauma in a naturalistic setting (at home) would reduce the occurrence of intrusive memories over the following week (ii) That any effect of sleep deprivation on intruisve memories is not merely the result of a shift in their time course (iii) That sleep deprivation may selectively reduce intrusive (involuntary) memories without impacting voluntary memory of the trauma film.

## Materials and methods

### Participants

Fifty-one healthy young adults were recruited to the study and randomised following the trauma film to two groups: sleep deprivation (*n* = 27) or sleep as usual (*n* = 24); one participant was excluded from the sleep-deprived group due to insuffient polysomnographic (PSG) or actigraphic records for the whole of the sleep deprivation period. The final sample included in the analysis was therefore 50 participants: sleep deprivation group, *n* = 26, 14 females, mean age = 25.16 ± 5.23 years; and sleep group, *n* = 24, 13 females, mean age = 23.12 ± 2.10 years. The participants were non-smokers; had no mental health-, alcohol- or drug-abuse problems (Mini International Neuropsychological Interview, MINI^31^); did not have a definitive morning or evening chronotype (Morningness–Eveningness Questionnaire, MEQ^32^; score of <69 or >31); did not have self-reported poor sleep (Pittsburgh Sleep Quality Index, PSQI^33^; score of <5); were not taking any medication (except for the contraceptive pill); and none of the female participants were pregnant. None of the participants had previously taken part in another research study using similar film footage and all participants provided written informed consent and received an honorarium for their participation. The study was approved by the National Research Ethics Service (NRES) committee, East of England, Hatfield (REC number 14/EE/0186).

### Procedure

**Fig. 1:**
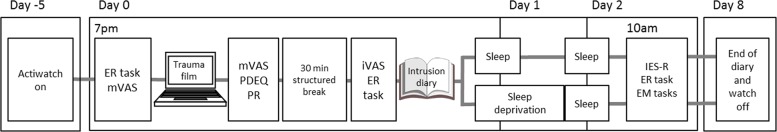
Study protocol. Participants wore an actiwatch for 5 days before the experimental session. In the evening of day 0, the participants were shown the trauma film. Before watching the film, the participants completed a mood visual analogue scale (mVAS) and the emotional response (ER) task. Immediately following the trauma film, participants completed the mVAS again as well as the peritraumatic dissociative experiences questionnaire (PDEQ) and personal relevance questions (PR). After a 30-min structured break, participants rated their initial experience of intrusive memories of the film (using a visual analogue scale, iVAS) and completed the ER task again. Participants were then instructed how to fill in the intrusion diary, in which they recorded the number of intrusive memories of the film daily and then randomised to either the sleep-deprived (asked to remain awake from the time of the film on day 0 to the end of day 1) or the sleep group (allowed to sleep at the end of day 0), set up with polysomnography (PSG), and returned home. On day 2, once all participants had had at least one night of sleep they returned to the lab to complete the ER task again, as well as explicit memory tasks (EM tasks)

Each participant wore an actigraphic device and completed a sleep diary for 5 days before viewing the trauma film (and for the rest of the study), to ensure good sleep history before the study (Fig. 1). On the day of the trauma film (day 0), participants reported to the study centre at 6–7 pm. Before the trauma film, participants provided fear ratings to images from the trauma film (emotional response task, session 1: before film, more details provided below) and rated their mood (using a series of visual analogue scales: mood VAS, more details provided below). The participants then watched the trauma film under controlled conditions as described below. Straight after the film, participants completed the mood VAS again, as well as questionnaires assessing state dissociation (peritraumatic dissociative experiences questionnaire, PDEQ^34^), personal relevance of the film clips watched and attention paid to the film. Participants completed the second session of the emotional response task 30 min after the trauma film (after the film), a break which included a series of standardised tasks so that the amount of time spent doing various activities in the break (e.g. visuospatial or verbal in nature) did not differ between participants. Participants were then instructed how to complete the pen and paper intrusion diary for the rest of that day (day 0) and the subsequent 6 days (first week) and completed an initial assessment of intrusive memories using a VAS. Participants were then randomised to either the sleep deprivation or sleep group, using a minimisation protocol ensuring gender balance between groups^35^. After PSG set-up, participants returned home. PSG equipment was removed the next morning to allow participants to return to their normal daily routine. On day 2, after the recovery sleep for the sleep-deprived group, and the second night of sleep for the sleep group, the participants returned to the study centre at 10–11 am to complete the final session of the emotional response task as well as the explicit memory tasks and the IES-R^21^. At the end of the study, participants returned the actigraphic device and study diary, which was checked by a researcher for completeness and legibility.

### Film with traumatic content (trauma film)

Participants viewed a film with content designed to relate to real experiences of trauma antecedent to PTSD (and referred to in its diagnostic criteria) such as scenes of a car crash, self-harm and the aftermath of genocide. The use of traumatic film footage has long been used as analogue of exposure to psychological stress^36^. The DSM-5 now allows exposure to trauma via visual material when it is in the line of work, e.g. a police officer who reviewed the horrific film footage of a homicide. This suggests that clinically significant post-traumatic symptoms can arise after a traumatic event that has been experienced indirectly, such as via film footage^37^, and supports the ecological validity of using aversive film material in the laboratory^19^. The trauma film used in this study was a 15-min compilation of 11 traumatic and distressing clips^26,38^. Participants were instructed to imagine that they were there at the scene watching the events unfold in front of their eyes, to give the film their full attention, not to close their eyes or look away. The film was shown on a laptop computer with a 15-inch screen, with the lights turned off and the researcher not present in the room.

#### Sleep deprivation at home

Participants in the sleep deprivation group were required to keep themselves awake at home. During the PSG set-up, participants were also told how their sleep would be monitored, with emphasis on the fact that it would be possible to tell if they had slept or not. During the sleep deprivation period and for 3 days prior, all participants were asked to stop consuming caffeine and alcohol, to not watch any violent or scary films and not to play any violent or scary video games. Besides these, participants were free to choose their own activities. Participants were asked to complete a record of the activities undertaken, but unfortunatley, the data could not be analysed, as the majority of participants did not complete it. Participants also received an automated text message every hour with a link to a timed stamped online questionnaire, the data of which will be reported separately.

### Assessments

#### Intrusive memory diary

The intrusion diary consisted of a pen and paper diary used daily to (1) report all intrusive memories; (2) give detailed content of each intrusion, so that it can be verified as matching a scene in the trauma film; (3) rate the level of distress experienced with the intrusion from 0 (not at all) to 10 (extremely); and (4) confirm the intrusion-involved mental imagery. Intrusive memories were defined as spontaneously occurring (i.e., not deliberately or effortfully retrieved) image-based memories of the trauma film, which can be in any mental imagery modality (visual and/or auditory) for example the sight of a red car or the sound of screaming. Participants were given standardised verbal and written instructions about the nature of intrusive memories. All intrusion diaries were second scored by EJ, who was blinded to the groups. Any discrepancies were discussed by KP and EJ until a consensus was reached. The frequency of intrusive memories was calculated for each day as the total number of image-based intrusions relating to the trauma film. For each intrusive memory experienced, participants also rated how distressing it was from 0 (not at all) to 10 (extremely). Since participants experienced different numbers of intrusive memories, proportional distress ratings relative to the number of intrusive memories experienced were calculated for each participant per day: sum of intrusive memory distress and number of intrusive memories.

#### Explicit memory tasks

A visual recognition task involved participants rating on paper if they recognised neutral images presented for 5 s as being from the film (OLD, 11 images) or not (NEW, 11 images)^26^. These neutral images from the film were different from those used in the emotional response task. Recognition performance was calculated as percentage of correct responses for both NEW and OLD images. For the verbal cued recall task, participants rated if 32 written statements relating to the trauma film were true or false. Recall performance was calculated as percentage of correct responses for both TRUE and FALSE statements.

#### Emotional responses to film stills

Emotional responses to film stills were assessed using a task based on Kuriyama et al.^30^. Participants rated fearfulness using a VAS for neutral images (salience confirmed in a separate population, *n* = 11) from the trauma film (1 image per clip) and for those not from the trauma film but of similar content. The images were presented for 7 s in a randomised order. Participants completed the same task immediately before the trauma film (before film), 30 min (after film) and ~36 h after the trauma film (after sleep). Mean fear ratings were calculated for the old and new images for each time point.

#### Sleep monitoring

Participants wore an actigraphic device (Actiwatch AWL, CamNtech, Ltd., Cambridge, UK) on their non-dominant wrist and kept a standardised diary of bed times for 5 days before viewing the trauma film until the end of the study (7 days after watching the trauma film). Actigraphy data were sampled in 1-min epochs and analysed with MotionWare software (version 1.1.15, CamNtech, Ltd.). Rest–activity patterns were manually entered and “Sleep start” and “Sleep end” calculated automatically using medium sensitivity. On the first night following the trauma film, all participants wore PSG. The Actiwave system (CamNtech Ltd., Cambridge, UK) was used with a nine-electrode montage: Fz:A2; C3:A2; Pz:A2; Oz:A2 (recorded using an EEG/ECG 4 unit), C4:A1 (recorded using an EEG/ECG 1 unit), EOG1:A1; EOG2:A1 (recorded using an EEG/ECG 2 unit) and EMG1:chin; EMG2:chin (recorded using an EMG 2 unit). The EEG and EOG channels had a sampling rate of 128 Hz and EMG channels had a sampling rate of 256 Hz. The data were scored for 30-s epochs according to the American Academy of Sleep Medicine (AASM) criteria. All PSG recordings were scored by KP and second scored blinded to the study group by RS with a mean concordance rating of 95% agreement.

Sleep timings were determined by PSG or actigraphy. PSG recordings were not adequate to score the whole night for five participants (two sleep-deprived and three sleep), so actigraphy recordings were used. For PSG recordings, sleep was defined as 5 min of continuous-stage NREM2, NREM3 or REM and a period of wake as 5 min of continuous active wake, including eye blinks, more than 50% alpha, increased EMG and movement. For actigraphy recordings, in this study 30 min of no or little activity was classed as a sleep episode and wake as more than 10 min of activity during a sleep episode.

#### Structured break

Participants completed 10 min of a verbal task that involved answering general knowledge questions with the aid of a reference book^39^, 10 min rating the pleasantness of music clips, followed by another 10 min completing the verbal task with different questions. The purpose of these tasks was to engage the participants in the same tasks for the 30-min break, not to assess the outcomes of the tasks.

#### Questionnaires

Mood was assessed before and after the trauma film using a 100-mm VAS for sadness, hopelessness and depression anchored to ‘not at all’ and ‘extremely’. A composite negative affect score was calculated by averaging scores across all scales and the change in negative affect was calculated across the trauma film (after film negative affect–before film negative affect). After viewing the film, self-reported attention paid to the film and personal relevance to the film clips were assessed using 10-point scales. State dissociation (i.e. the level of detachment from reality) was also assessed following the trauma film using the PDEQ^34^, a 10-item scale with a score range of 10–50. The intrusion VAS asked ‘since watching the film, to what extent have mental images of the film sprung to mind?’ anchored by ‘not at all’ and ‘very much’, and scored 0–100. The intrusion sub-scale of the IES-R^21^ was used, giving a score in the range of 0–4. In line with previous research, the trauma film was used as an index event^38^.

### Statistical analysis

All statistical analyses were assisted by a statistician and performed using R version 3.0.1 (16 May 2013) (Copyright (C) 2013, The R Foundation for Statistical Computing). Sample size was calculated to attain an effect size of 0.54, with a power of 0.90 and an alpha of 0.05 between groups, based on our previous study^20^. Simple group comparisons of continuous variables were performed using the independent *t*-test for normally distributed data, or the Mann–Whitney for non-normally distributed data. Group comparisons of categorical data (e.g. gender) were performed using the Pearson chi-square test. Data on a fixed scale, i.e. from a questionnaire or VAS with a fixed minimum and maximum score that did not satisfy checks for normal distribution or had ties of data (multiple data points of the same value), were transformed using a folded logarithmic transformation, where the data are distributed on a log scale rather than the fixed minimum and maximum score: log(*x* + (minimum score + 1))/((maximum score + 1) *−* *x*), where *x* = the raw data. Before-film and after-film negative affect scores were analysed with an analysis of variance (ANOVA), as these data fitted a normal distribution. A linear mixed effects model was used to analyse the change in fear ratings in the emotional responses to film still task, using the R-package nlme. Intrusive memory data were analysed as week total from the day after the trauma film (day 1) to the end of the study (day 6). Intrusions were then assessed daily from the day after the trauma film (day 1 to day 6). In addition, the numbers of intrusive memories reported on the day of the trauma film (day 0) were assessed between groups. Importantly, day 0 is longer on average for the sleep-deprived group compared to the sleep group as this day includes the sleep deprivation period. On examining the number of intrusive memories reported for both conditions (sleep deprivation versus sleep) separately using box plots, two participants reported a number of intrusive memories more than three standard deviations above the mean (1 per condition). Therefore, all analyses of intrusive memories were conducted both with and without these participants in order to examine their influence. Results of the analyses with these participants are reported and only divergent findings from the analyses without these participants are reported. Intrusive memories were fitted to a two-level generalised linear model using a Poisson regression with the R-package lme4. All statistics are reported as mean and standard deviation unless stated.

## Results

### Baseline participant characteristics

Before the trauma film, participants had a good sleep history (actigraphy sleep duration of 6.65 h ± 1.09; PSQI = 3.36 ± 1.47) and intermediate chronotype (MEQ = 53.62 ± 8.04), with no statistical difference seen between the groups (Table 1). Participants showed an increase in negative affect after watching the trauma film, which was found not to be statistically different between groups (Change in negative affect: sleep deprived = 13.31 ± 16.19, sleep = 10.91 ± 17.72, ANOVA: film effect: *F* = 25.50, *P* < 0.001, group/film interaction: *F* = 0.25, *P* = 0.62). After watching the trauma film, the groups experienced no difference in state dissociation (PDEQ: sleep deprived = 15.15 ± 5.79, sleep = 13.71, 5.44, Mann–Whitney: *Z* = −1.53, *P* = 0.13); personal relevance to the film clips used (sleep deprived = 4.42 ± 2.76, Sleep = 3.38 ± 2.55, *T*-test; *t* = 1.39, *P* = 0.17); or attention paid to the film (sleep deprived = 9.00 ± 1.10, Sleep = 9.21 ± 0.72, *T*-test: *t* = −0.66, *p* = 0.51). An initial assessment of intrusive memories experienced in the first 30 min after the trauma film (before group randomisation) using an intrusion VAS found no difference between the groups (sleep deprived = 45.08 ± 23.60, sleep = 40.25 ± 23.54, Mann–Whitney: *Z* = −0.61, *p* = 0.54).

**Table 1:** Baseline participant characteristics

	Sleep deprived (*n* = 26)		Sleep (*n* = 24)		Test value	*p*
	Mean	SD	Mean	SD		
Age	25.16	5.23	23.12	2.1	1.75	0.087
Gender	14		13		1.287	0.526
MEQ	54.81	7.92	52.33	8.13	1.09	0.281
PSQI	3.31	1.38	3.42	1.59	−2.06	0.796
*Actigraphy*						
Bedtime	0.45	1.07	0.50	0.99	−0.44	0.663
Get-up time	8.39	0.80	8.36	0.79	0.13	0.897
Time in bed	7.95	0.83	7.86	0.77	−0.22	0.823
Sleep efficiency	82.86	8.20	81.15	9.63	−0.49	0.624
Sleep latency	0.13	0.15	0.15	0.17	−1.07	0.286

### Sleep deprivation

**Fig. 2:**
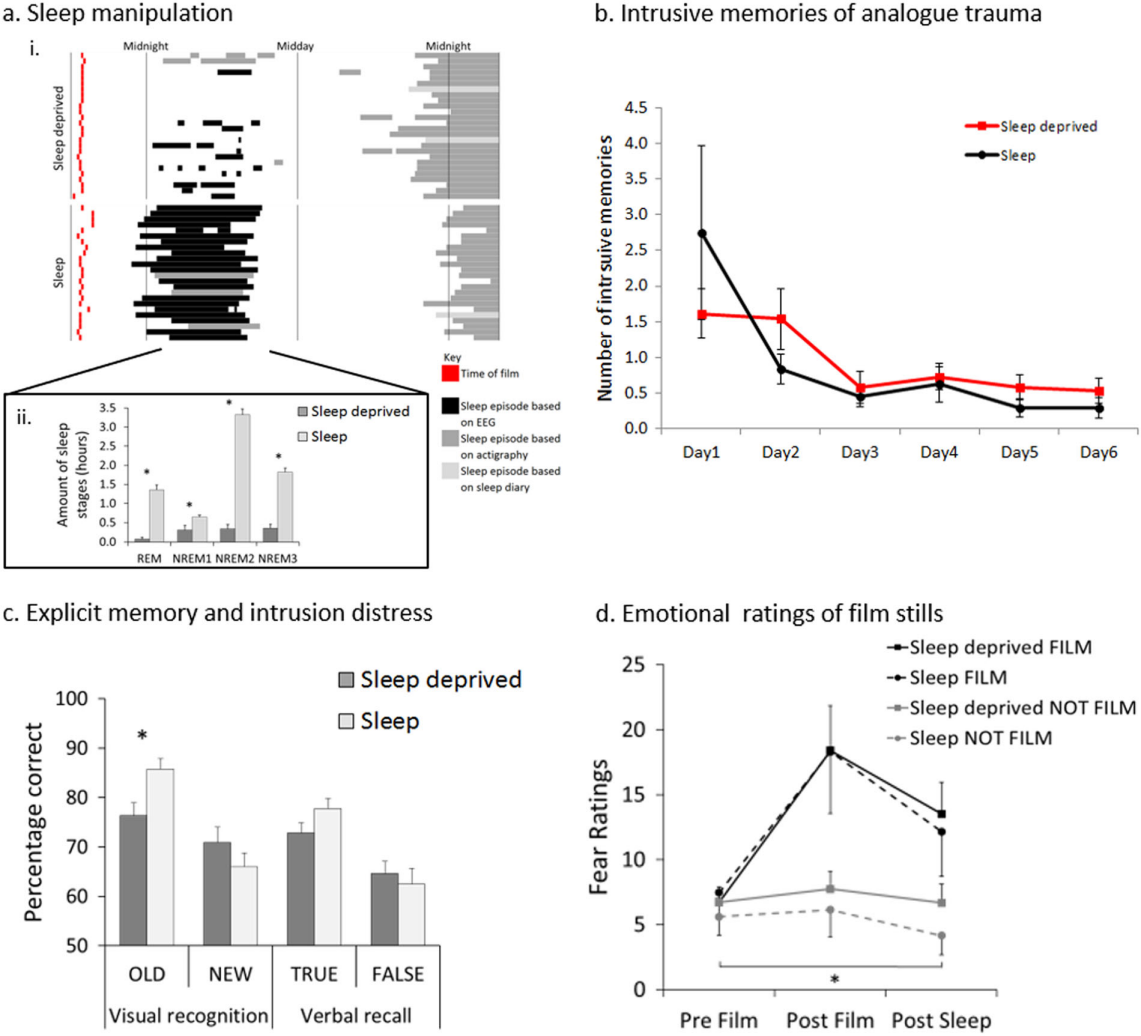
Experimental findings. **a** Sleep manipulation achieved. Sleep deprivation at home was successful, with the sleep-deprived group showing significantly less sleep than the sleep group: (i) The time each participant watched the trauma film (red) is shown along with the times they slept during the subsequent 24 h as assessed by polysomnography (black), actigraphy (dark grey) and sleep diary (light grey). (ii) Mean duration of sleep stages. **b** Intrusive memories of analogue trauma. Number of intrusive memories reported for the sleep-deprived participants (red line), compared to the sleep group (black line) for each day after the trauma film. **c** Emotional ratings of film stills. Mean fear rating of stills from the study film (FILM, black) and stills not from the film (NOT FILM, grey). **d** Explicit memory and intrusion distress. Mean percentage of correct responses for old (OLD) and new (NEW) images from the study film in the visual recognition task and true (TRUE) and false (FALSE) statements about the study in the verbal recall task. All error bars = standard error of the mean, * ≤ 0.01

The sleep-deprived participants slept on average 1.49 ± 1.77 h, compared to 7.22 ± 1.43 h for the sleep group, in the first 24 h following the trauma film (*T*-test: *t* = −10.16, *p* < 0.001, Fig. 2a.i.), and had significantly less REM and NREM sleep (*T*-test: REM *t* = −9.63, *p* < 0.001; NREM1 *t* = −2.70, *p* = 0.01; NREM2 *t* = −15.71, *p* < 0.001, NREM3 *t* = −10.19, *p* < 0.001, Fig. 2a, ii). Of the sleep-deprived participants, nine remained completely awake for the whole sleep deprivation period. The remaining 14 participants slept between 0.17 and 6 h and had between one and four sleep bouts. All participants were included in the primary analysis; however, post-hoc analysis was performed to address the impact of the heterogeneous sleep profiles in the ‘sleep-deprived’ group.

### Intrusive memories to the trauma film during the first week (days 1–6)

Overall, 369 intrusive memories were reported by participants; of those 96.75% were matched to content from the trauma film, resulting in 357 intrusive memories included in the analysis. In all, 42.3% of intrusive memories were of film clips depicting scenes of road traffic collisions, 17.37% were of a film clip involving self-mutilation (facial cutting), 13.73% of news footage of an elephant trampling people, 12.61% of scenes of drowning, 9.53% of footage of surgery and 4.48% of news footage of the Rwanda genocide.

No difference was found in the total number of intrusive memories reported between the two groups during the first week (days 1–6) following the trauma film and sleep manipulation (sleep deprived = 5.58 ± 6.14; sleep = 5.25 ± 7.05, z = −0.50, *P* = 0.62, Table 2). Daily analysis of intrusive memories showed statistically significant differences between the two groups in the number of intrusive memories reported on day 1 and day 2 after the trauma film (Fig. 2b, Table 2). On day 1, the sleep-deprived participants reported statistically fewer intrusive memories compared to the sleep group (sleep deprived = 1.62 ± 1.79; sleep = 2.75 ± 5.97, *z* = 2.70, *P* = 0.01). In contrast, on day 2 the sleep-deprived group reported statistically more intrusive memories (sleep deprived = 1.54 ± 2.16; sleep = 0.83 ± 1.01, *z* = −2.24, *p* = 0.03). However, these group differences were driven by two participants who reported high numbers of intrusive memories (Table 2).

**Table 2:** Intrusive memories

	Sleep deprived		Sleep					
	Mean	SD	Mean	SD	Estimate	Std. error	*z* value	*p* value
Day 1	1.62	1.79	2.75	5.97	0.53	0.20	2.70	0.01
Day 2	1.54	2.16	0.83	1.01	−0.61	0.27	−2.24	0.03
Day 3	0.58	1.14	0.46	0.72	−0.23	0.40	−0.58	0.56
Day 4	0.73	0.92	0.63	1.21	−0.16	0.35	−0.45	0.65
Day 5	0.58	0.90	0.29	0.62	−0.68	0.46	−1.49	0.14
Day 6	0.54	0.90	0.29	0.69	−0.61	0.46	−1.32	0.19
Total	5.58	6.14	5.25	7.05	−0.06	0.12	−0.50	0.62
*Sensitivity analysis*								
Day 1	1.48	1.69	1.57	1.44	0.06	0.23	0.24	0.81
Day 2	1.20	1.32	0.74	0.92	−0.48	0.30	−1.60	0.11
Day 3	0.52	1.12	0.48	0.73	−0.08	0.41	−0.20	0.84
Day 4	0.64	0.81	0.65	1.23	0.02	0.36	0.05	0.96
Day 5	0.48	0.77	0.30	0.63	−0.46	0.48	−0.96	0.34
Day 6	0.40	0.58	0.30	0.70	−0.27	0.49	−0.56	0.58
Total	4.72	4.40	4.04	3.94	−0.15	0.14	−1.12	0.27

Because it was reasonable to assume that participants either failing to sleep deprive or failing to sleep adequately did not properly belong in their respective groups, post-hoc analysis was performed with participants in the sleep-deprived group who slept more than 10 min (*n* = 14) and participants in the sleep group who slept less than 6 h (*n* = 1) removed. The subsequent analysis found that the total sleep-deprived group (*n* = 12) reported more intrusive memories than the total sleep group (*n* = 23): sleep deprived = 9.50 ± 9.66; sleep = 6.96 ± 8.82, *z* = −2.54, *P* = 0.01.

**Table 3:** Linear mixed effect model of emotional responses to film stills and control images

	Film				Not Film			
	Coeff.	df	*t*	*p*	Coeff.	df	*t*	*p*
Group	−0.09	48	−0.34	0.734	−0.28	48	−1.21	0.233
Post film	0.97	94	5.07	<0.001	0.14	94	1.27	0.206
Post sleep	0.54	94	3.55	<0.001	−0.08	94	−0.78	0.436
Post film:Group	−0.24	94	−0.86	0.393	−0.24	94	−1.45	0.152
Post sleep:Group	−0.27	94	−1.22	0.227	−0.27	94	−1.80	0.076

### Immediate intrusive memories to the trauma film (day 0)

The number of intrusive memories reported immediately after trauma film viewing (i.e. the evening and night after trauma film viewing, day 0) was not statistically significant between the two groups (sleep deprived = 1.62 ± 1.86; sleep = 1.83 ± 2.33, *z* = 0.59, *p* = 0.56), despite this time period being of different durations between the groups (sleep deprived = 16.90 ± 8.87 h; sleep = 5.73 ± 1.35 h).

### Intrusive memory distress

No difference was found between the groups for the intrusion subscale of the IES-R (sleep deprived = 0.71 ± 0.57, sleep = 0.61 ± 0.45, *t* = 0.68, *p* = 0.50). The average distress caused by an intrusive memory (relative to the number of intrusive memories experienced) was also similar between groups (sleep deprived = 1.93 ± 1.94; sleep = 2.32 ± 2.32, *t* = 0.48, *p* = 0.64).

### Explicit memory of the trauma film

For the visual recognition task completed on day 2 (Fig. 2c), the sleep-deprived participants remembered significantly fewer of the images from the study film (OLD) compared to the sleep group (sleep deprived = 76.37 ± 12.86; sleep = 85.77 ± 10.19, *t* = −2.82, *p* = 0.01). No group difference was found for the number of new images (NEW) correctly identified as being new (sleep deprived = 70.91 ± 15.53; sleep = 66.01 ± 13.21; *t* = −1.18, *p* = 0.24). In the cued recall task, also completed on day 2 (Fig. 2c), there was no difference between the groups in the number of correct responses to both true and false statements (TRUE: sleep deprived = 72.85 ± 10.13, sleep = 77.70 ± 10.19, *t* = −1.69, *p* = 0.10. FALSE: sleep-deprived = 64.62 ± 12.76, sleep = 62.50 ± 15.46, *t* = 0.53, *p* = 0.60).

### Fear ratings for images from the trauma film

Fear ratings were assessed for images taken from the trauma film and images of similar content, but not from the trauma film. A linear mixed effects model was used to assess the change in fear ratings from before film viewing to immediately after film viewing (after half an hour) and after at least one night of sleep (day 2). In both groups, fear ratings increased from before to after film viewing for images from the trauma film (Fig. 2d, Table 3). Elevated fear ratings persisted post sleep (day 2) once the participants had had at least one night of sleep, with no difference found between groups. For images that were not from the film, no change in fear ratings was found from before film viewing to after, for either group (Fig. 2d, Table 3). From before film viewing to after sleep, the sleep group experienced a significant decrease in fear ratings (*t* = −3.24, *p* = 0.002, Table 4). By contrast the sleep-deprived group showed no change (*t* = −0.78, *p* = 0.436, Table 4). However, this only resulted in a trend for a group interaction after sleep (*t* = −1.80, *p* = 0.076, Table 3).

**Table 4:** Linear mixed effect model of emotional responses to control images not from the film (as in Table 3) relative to sleep-deprived group and sleep group

	Relative to sleep-deprived group				Relative to sleep group			
	Coeff.	df	*t*	*p*	coeff.	df	*t*	*p*
Group	−0.28	48	−1.21	0.233	0.28	48	1.21	0.233
Post film	0.14	94	1.27	0.206	−0.09	94	−0.78	0.439
Post sleep	−0.08	94	−0.78	0.436	−0.36	94	−3.24	0.002
Post film:Group	−0.24	94	−1.45	0.152	0.24	94	1.45	0.152
Post sleep:Group	−0.27	94	−1.80	0.076	0.27	94	1.80	0.076

## Discussion

In this study, inconsistent sleep deprivation was achieved in participants’ home settings on the first night after experimental trauma. No difference was found in the number of intrusive memories reported over the week following the analogue trauma between the sleep-deprived versus sleep as usual conditions. Although inconsistent sleep deprivation at home resulted in fewer intrusive memories on day 1 after the trauma film, the opposite was found for day 2. Moreover, these statistical differences did not remain following removal of two individuals who reported high levels of intrusive memories. Thus, in this healthy young population, given the same experimental trauma but a difference between studies being inconsistent sleep deprivation at home (naturalistic setting) rather than total sleep deprivation in a hospital setting, we did not replicate the expected pattern of results of the frequency of intrusive memories^20^. Considering the conditions under which sleep deprivation (naturalistic versus hospitals) was achieved requires further examination.

Sleep as a target for the prevention of the occurrence of intrusive memories after an aversive event has to date received little attention, and early findings are mixed. Previously we have shown that, in a hospital setting, individuals who were totally sleep deprived on the first night immediately following an analogue trauma reported fewer intrusive memories over the subsequent week, driven by fewer intrusive memories on the first two days, compared to a control sleep as usual group^20,40^. In contrast, using a similar study design, Kleim et al.^41^ reported that women who *slept* as usual, compared to at least 8 h of wake (during the day or night), following a trauma film experienced *fewer* intrusive memories towards the end of the subsequent week (days 3, 5, 6 and 7). A nap study assessing the effect of sleep on cognitive bias modification-appraisal training found that healthy individuals who had a 90-min nap following a trauma film reported fewer intrusive memories, compared to a wake group, irrespective of appraisal training^42^. Interestingly, in the current study, the small number of individuals (*n* = 12) who achieved total sleep deprivation at home (slept less than 10 min, as assessed by PSG) also reported *more* intrusive memories over the week as a whole (7 days), compared to the sleep as usual group—i.e. the opposite of what we originally predicted. Although this finding must be considered tentatively as this post-hoc analysis using this subpopulation was greatly reduced in power, it is consistent with the Kleim^41^ and Woud^42^ studies, suggesting that prolonged wake can result in more intrusive memories being reported, compared to a period of sleep.

One of the limitations of the current study is the modest sample size. The small sample sizes in most experimental studies could be an explanation for these divergent findings and restricted conclusions, and this will be important to consider in designing future studies. Another possibility for the discrepancy between studies is methodological differences, in particular the conditions under which sleep deprivation (or wake) was achieved. In our previous study, participants were sleep deprived in a highly controlled hospital environment (constant light levels, regular snacks, no computer, television or music) and continually monitored by a researcher in the same room for 24 h after watching the analogue trauma. Moreover, the trauma film was shown in the same room as the participants were sleep deprived. As a result, the participants would have been exposed to the same contextual cues, as those experienced when the film was watched, throughout the sleep deprivation period, which could have resulted in habituation to trauma stimuli and hence fewer intrusive memories. By comparison, in the current study participants returned home after watching the trauma film, and in this naturalistic environment there were no constraints on light levels or activities (apart from not watching scary or violent films), which importantly included going about their normal daily routine during the day. In the Kleim study^41^, the conditions under which sleep deprivation was achieved appear not to be described, but, importantly, this group included two conditions (sleep deprivation at night and at least 8 h of wake during the day) and during the 8 h of wake during the day it is likely that they returned to their normal routine. Both the sleep and wake groups, in the Woud study^42^, remained in the testing facilities after film viewing, but after the 90-min nap or wake period they returned home to their normal routine. Thus, it is possible that the ameliorating effect of sleep deprivation we previously found could be dependent on the highly controlled conditions in conjunction with a total lack of sleep. Further research is needed to explore whether it may be the conditions individuals are exposed to following an analogue trauma (including context), rather than the extent of sleep deprivation, that could influence the frequency of intrusive memories. Moreover, the role of sleep in the timescale of intrusive memories following an analogue trauamtic event requires further consideration with our current and past studies finding a difference in the number of intrusive memories in the first few days and the Kliem study finding differences emerging towards the end of the first week^41^.

Sleep as a target for weakening other post-traumatic symptomatology, beyond just intrusive memories of the trauma, has also received attention. Kuriyama et al.^30^ found that sleep deprivation following a series of road traffic films ending with or without a collision reduced fear responses (fear ratings and change in skin conductance) to images from neutral films (without a collision). This reduction of fear responses to non-aversive film reminders suggests that sleep deprivation facilitates the extinction of generalised fear responses, i.e. fear responses to non-trauma-related cues. Clinically, over-generalization of fear reactivity from conditioned to non-conditioned stimuli has been reported for PTSD patients^43^, meaning individuals experience the same stress reactivity to safe stimuli (e.g. door slamming) that share features with the danger stimuli (e.g. gunshot). Therefore, preventing this over-generalization could be of clinical significance. Using an aversive and neutral film, Werner et al.^44^ assessed changes in emotional responses to film scenes after a night of sleep. Enhanced responses to aversive film reminders (increased electrodermal activity and reduced facial corrugator muscle reactivity) were correlated to longer durations of REM sleep the preceding night, suggesting REM sleep may attenuate emotional processing.

Clearly, at present results in experimental studies with humans are mixed. In animal models, PTSD-like behaviours, such as freezing behaviour to trauma cues and anxiety behaviour on the elevate plus maze task, are attenuated by total sleep deprivation during the first sleep phase (i.e. during the day for nocturnal animals) compared to sleep controls^45,46^, or correlated to lower amounts of REM sleep^47^. The translation of research between animal models and human experimental studies is critical for investigating the conditions under which sleep deprivation attenuates emotional responses to trauma cues. Moreover, how these findings relate to the role of sleep in non-trauma related emotional memory requires consideration. The emotional component of an emotional memory has been proposed to be downgraded by REM sleep, while the declarative core is consolidated^48–52^, however a body of work suggests that REM sleep enhances the emotional component^44,53–55^. Furthermore, several studies have found no effect of sleep on emotional responses to reminders of the emotional stimuli^14,56^. Sleep deprivation resulting in the attenuation of trauma-related responses, including the animal studies described above^45–47^ and our previous experimental study^20^, supports the theory of sleep, especially REM sleep, enhancement of the emotional salience of stimuli^14,53^. Whereas, the hypothesis that REM sleep degrades the emotional component is supported by sleep resulting in fewer intrusive memories to an analogue trauma^41–42^. The secondary aim of this study was to investigate the effect of sleep deprivation both on the time course of intrusive memories and on the voluntary memory of the trauma film including emotional response to trauma film cues. Despite the sleep-deprived participants being awake on average 11.2 h longer than the sleep group immediately following the trauma film, they reported only marginally (and not statistically significantly) more intrusive memories during this period (i.e. the period during which sleep deprivation occurred, day 0). Thus, the experience of intrusive memories in the sleep-deprived group was not simply shifted to an earlier time (i.e. while they were awake overnight). Thus, any effect of sleep deprivation appears not to be due to an extended period of time enabling more intrusive memoires to be experienced immediately after the trauma film, compared to the sleep group.

The emotional responses to trauma film cues were assessed using fear ratings for images from the trauma film, compared to images of a similar content not from the trauma film, a task based on that used by Kuriyama et al.^30^. Our findings show no effect of inconsistent sleep deprivation on emotional responses (i.e. fear ratings) to images that were from the trauma film. However, we did find that the sleep group had lower fear ratings for images not from the film. This finding must be treated with caution as it was only shown in the within-group analyses and remained only as a trend in the between-group comparisons. By contrast, Kuriyama et al.^30^ found that totally sleep-deprived individuals showed a quicker reduction (by day 3 compared to day 10 post film viewing) of fear responses to images not associated with an aversive event (road traffic footage ending without a collision—‘safe’ images) compared to a control sleep group. Although this initially appears contradictory to our findings, importantly in the Kuriyama study, both the sleep-deprived and sleep groups did show an initial increase in fear ratings for these ‘safe’ images following film viewing. This increase in fear ratings for non-aversive associated images (images not from the trauma film) was not seen in our study. A key difference between the studies is the use of images from the same kind of film footage (road traffic scenes) associated with or without an aversive event (traffic collision) in the Kuriyama study, compared to our study where images were either from the film or not from the film, but of a related content. Therefore, the ‘safe’ images in our study may not have been an adequate equivalent to those in the Kuriyama study.

To assess the impact of sleep deprivation on the voluntary memory of the trauma film, explicit memory for the trauma film was assessed using a visual recognition task and verbal cued recall task. Explicit recognition of images from the trauma film (although not verbal cues) appears consistent with the mostly positive effect of sleep on emtional memory consolidation^17,27,28,57^, as the sleep-deprived participants remembered significantly fewer images compared to the sleep group. Therefore, inconsistent sleep deprivation at home does appear to weaken (if only marginally) the consolidation of voluntary memory of the trauma film. This could provide an explanation for fewer intrusive memories being reported, i.e. all memory of the trauma film is weakened by sleep deprivation. Although it is a weak finding, further research could examine if changes in voluntary memory due to sleep deprivation are related to changes in involuntary intrusive memories^58^.

## Limitations

Further work is clearly needed to determine the role that the first episode of sleep after a traumatic event plays in the memory of that event and subsequent psychological reactions—such as the impact on intrusive memories and other post-traumatic symptomatology. Any analogue study design is limited in its capacity to generalise its findings to real-world situations. The experience of a real-life trauma is also more distressing and complex than anything we can, and would want to, simulate in the lab. In the real world, sleep disturbances have been reported to be *predictive* for the development of PTSD, as well as other psychiatric disorders, when self-reported both before^22^ and after^23^ a traumatic event and when objectively assessed 1 month following trauma^24,25^. Prospective assessment of sleep from the moment of trauma in the real world is needed to determine if sleep immediately following a trauma has a differential role in the development of post-traumatic symptomatology compared to disturbed sleep in the subsequent weeks and months. However, sleep in the aftermath of a real-life trauma has only been investigated in the weeks and months following a trauma event or has been reliant on retrospective accounts of sleep history. Therefore, the impact of a traumatic event on the first episode of sleep immediately after that event and the impact on short- and long-term psychopathology is also needed to determine the beneficial role, or otherwise, of sleep on the day of trauma.

Although consciously undertaken to create a more ecologically valid study design, consequential trade-offs must be considered for the loss of experimental control by taking participants out of the lab and into the home environment. Participants were monitored at home using polysomnography and actigraphy, but were not otherwise observed. Thus, it is possible that they consumed caffeine during this period (although they were asked to abstain for 3 days before viewing the trauma film and throughout the sleep deprivation period). The scheduled drug screening in the morning will undoubtedly have demotivated most participants to consume any prohibited substances. Participants were also able to undertake any activities they wished during this period, which could influence the frequency of intrusive memories. Attention to the trauma film itself and its emotional impact will vary between individuals, also adding to the heterogeneity within this sample. Furthermore, the ecological validity of sleep deprivation at home compared to in a hospital setting following a traumatic event (if indeed it is ever adopted) may well depend on the type and circumstances of the traumatic event. For example, only around 30% of individuals were admitted to hospital overnight following a road traffic collision in a recent clinical trial^59^. Indeed, keeping people in hospital overnight might be easier to implement as the treatment of choice if, indeed, post-trauma sleep deprivation was ever to be adopted. The number of intrusive memories reported in experimental studies, including this study, can be lower compared to real-life situations, somewhat hampering the clinical (and general) meaning of the results. However, we also note that for a diagnosis of PTSD for example, patients only need to experience two or more intrusive memories every month (with distress clearly present), reflecting that even at relatively low frequency intrusive memories can carry clinical significance and lead to impairement in functioning. Future research could seek to enhance the generation of intrusive memories in the lab by for example using longer or more distressing film clips. Finally, achieving total sleep deprivation at home was not possible in all participants in this study, the costs and benefits of such an approach must be considered for future research.

In conclusion, in this study we have found that inconsistent sleep deprivation at home on the first night following a trauma film does not result in the same reduction of intrusive memories as seen with total sleep deprivation in a hospital setting (nor in line with earlier animal models^45–47^). The first episode of sleep, however, remains an interesting candidate to explore to target intrusive memories as well as other post-traumatic symptomatology in the aftermath of a traumatic event.

## Data Availability

The research materials supporting this publication can be accessed by contacting the corresponding authors.
